# Wound Management Property of a Hydroethanolic Leaf Extract of *Cnestis ferruginea* DC

**DOI:** 10.1155/2021/6693718

**Published:** 2021-06-17

**Authors:** Jibira Yakubu, G. A. Koffuor, Talent Atsu-Nunyawu, Jeremiah Aboagye, Irene Aasam Aabeinir, Fasilatu Alhassan, Lord Christian Ocran, Philomena Entsie

**Affiliations:** ^1^Department of Pharmacology, Faculty of Pharmacy and Pharmaceutical Sciences, Kwame Nkrumah University of Science and Technology, Kumasi, Ghana; ^2^Department of Herbal Medicine, Faculty of Pharmacy and Pharmaceutical Sciences, Kwame Nkrumah University of Science and Technology, Kumasi, Ghana

## Abstract

**Objective:**

To establish the wound management property of a hydroethanolic *Cnestis ferruginea* leaf extract (CFHE).

**Materials and Methods:**

The wound area was measured after excision at the dorsal part of the Albino rats, and after treatment with 5–15% *w*/*w* CFHE ointments for 14 days. Absorbances of platelet-rich plasma treated with 0.8–100 mg/mL CFHE and an aggregating agent were spectrophotometrically determined in an *in vitro* platelet aggregation test. Wound tissue histopathology of CFHE ointment-treated animals revealed angiogenesis, reepithelialization, deposition of collagen, and granular tissue formation in wound tissues. Reduction in thigh oedema and pain threshold, in 7-day-old chicks, were assessed by carrageenan-induced oedema and Randall-Sellito pressure models, respectively. By the Agar diffusion method, bacterial growth inhibition by a 15% *w*/*w* CFHE ointment was investigated on *Salmonella typhi*, *Escherichia coli*, *Pseudomonas aeruginosa*, *Staphylococcus. Aureus*, *and Streptococcus pyogenes*.

**Results:**

All concentrations of CFHE ointment significantly reduced (*p* < 0.0001) wound area by 29–41% posttreatment. CFHE (1.6–100 mg/ml) promoted platelet aggregation (*p* ≤ 0.0001) by 37–67% (IC_50_: 3.1–6.2 mg/ml). There were improved wound tissue reepithelization, fibroblast proliferation, angiogenesis, and collagen deposition with 15% CFHE ointment treatment. CFHE ointment significantly (*p* ≤ 0.0001) and dose-dependently reduced thigh oedema and showed a significant (*p* ≤ 0.05) analgesic effect. *In vitro,* 15% CFHE ointment caused >100% growth inhibition of selected bacteria.

**Conclusion:**

The hydroethanolic leaf extract of *Cnestis ferruginea* possesses wound healing, platelet aggregation, anti-inflammatory, analgesic, and antimicrobial properties and, hence, could be effective in the management of open and some closed wounds.

## 1. Introduction

A wound is the discontinuity of the skin's epithelium because of an injury to the skin or its underlying tissues [1]. It may occur because of physical, chemical, thermal, microbial, or immunological attack on the tissue [2]. In the chronic state, intense pain, infection, loss of tissue or organ function, amputations, and death sometimes characterized wounds [3]. The prevalence of chronic wounds has been estimated to be between 0.18% and 1.3% in the aged population across the globe, and its treatment and/or management are estimated to cost more than $1 billion per year [4]. Globally, the annual review for the cost of treating wounds was about $2.8 billion in 2014, and it is projected to exceed $22 billion by 2024, driven by technological advancement, rising incidences of chronic wounds, increasing government support, and a rising geriatric population [5].

Wound management presents huge financial cost and significant undesirable socioeconomic effects, especially on individuals with other comorbidities. In Ghana, the situation of the inability of wounds to heal compounds by the exponential increase in the number of noncommunicable diseases such as diabetes mellitus and microbial infections especially in rural communities has been studied, where the prevalence and impact of chronic wounds are on the increase [6–9]. Wound management involves not only taking care of the complex physiological and dynamic process of wound healing, i.e., hemostasis phase, the inflammatory phase, the proliferation phase, and the remodeling phase [10], but also the relief of pain and the prevention of infections, as these hasten the healing process.

In Ghana, because of the high cost of orthodox medicines coupled with inadequate health care facilities and healthcare professionals especially in rural areas, most individuals rely on folkloric medicines, which are believed to be efficacious, readily available, affordable, and of low toxicity for management of wounds [11–13]. The widespread traditional uses of plants stimulate the scientific community to search and provide proof of efficacy for these wound management herbals [14]. *Cnestis ferruginea* DC (Family: Connaraceae), a popular plant in Ghana, with local names as *Apose* (Twi), *Akitase* (Fante), and *Pudaegye* (Nzema), is one such medicinal plant useful in a folkloric way in wound healing. This study, therefore, sought to investigate wound management activities of a hydroethanolic leaf extract of *Cnestis ferruginea* to reveal its usefulness to the health care community using *in vitro* and *in vivo* scientific experimental models.

## 2. Materials and Methods

### 2.1. Plant Collection

The fresh leaves of *Cnestis ferruginea* were collected from Ayigya, Kumasi, in the Ashanti Region of Ghana (latitude 6°35 N to 6°40 N and longitude 1°30 W to 1°35 W) in January 2020. Mr. Asare Sarfo, a Botanist at the Department of Herbal Medicine, KNUST, Kumasi, Ghana, did identification and authentication of the plant specimen.

### 2.2. Preparation of Plant Extract

The fresh leaves of *Cnestis ferruginea* were washed and shade-dried (Temperature: 25–34°C). The dried leaves were pulverized into a smooth powder using a hammer mill (Lab mill machine, Christy and Norris, Chelmsford, England). A 1 kg quantity of the powder was cold macerated using 5 l of 70% ethanol (to extract both aqueous and organic active principles in the plant material) for 72 hours with occasional stirring. The macerate was filtered using a muslin cloth (the residue was remacerated three times to get maximum yield). The filtrate obtained was concentrated using a rotary evaporator (Rotavapor BÜCHI R-200 with heating bath B-490, Büchi, Konstanz, Hamburg, Germany) and freeze-dried (YK-118 Vacuum Freeze Drier, True Ten Industrial Company, Taiwan) at Council for Scientific and Industrial Research-Forestry Research Institute of Ghana (CSIR-FORIG), Fumesua, Kumasi, Ghana. The extracted powder weighing 84.7 g (percentage yield: 8.47%) was labelled as *Cnestis ferruginea* hydroethanolic leaf extract (CFHE) and stored in a desiccator for this study.

### 2.3. Formulation of CFHE Ointment

CFHE ointments of concentrations 5, 10, and 15% *w*/*w* were formulated from simple ointment British Pharmacopoeia (BP) composed mainly of petroleum jelly [15]. To prepare these concentrations, each of 2.5, 5.0, and 7.5 g of the CFHE was mixed with 47.5, 45.0, and 42.5 g of the simple ointment BP, respectively, and heated gently at a temperature of 60ºC, while stirring continuously until a homogenous mixture was obtained. The preparation was then cooled, packaged, and labelled appropriately.

### 2.4. Ethical Considerations

Animal Ethical Committee (FPPS-AEC/CA01/13), Faculty of Pharmacy and Pharmaceutical Sciences, Kwame Nkrumah University of Science and Technology, Kumasi, Ghana, approved the animal studies.

### 2.5. Experimental Animals

Twenty-five (25) male adult albino rats weighing 150–200 g and 6–8 weeks of age obtained from the animal facility of the Department of Pharmacology, KNUST, Kumasi, Ghana, were used in the wound-healing study, while forty-seven-day-old Cockerels (*Gallus gallus*, strain Shaver 579, Akropong Farms, Kumasi, Ghana), weighing 30–50 g, were used in the anti-inflammatory and analgesic studies. The animals were kept in a metal wire gauze cages according to their predetermined groups. The floor of each cage was covered with coarse sawdust, under controlled environmental conditions (temperature at 25 ± 3ºC, relative humidity of air at 50–70%, and ambient light/dark cycle). Rats fed on a standard pellet diet (Agricare Ltd, Kwadaso, Kumasi) and cockerels with Chick Mash (Agricare Ltd, Kwadaso, Kumasi), and water was given *ad libitum* during the entire experimental period. The animals were kept and treated under the National Institute of Health Guidelines for the Care and Use of Laboratory Animals (NIH, Department of Health and Human Services publication no. 85–23, revised 1985).

### 2.6. Microorganisms Used in This Study

The clinical strain microorganisms used in the microbial susceptibility determination were *Escherichia coli, Pseudomonas aeruginosa, Staphylococcus aureus, Salmonella typhi,* and *Streptococcus pyogenes*. The microorganisms were obtained from the Department of Pharmaceutics and Microbiology laboratory, Faculty of Pharmacy and Pharmaceutical Sciences, KNUST, Kumasi, Ghana.

### 2.7. Preparation of Blood Samples Used in This Study

The sheep's whole blood was collected from Kumasi Abattoir Company Ltd, Asokwa, Kumasi, Ghana (Location: latitude 6°43′55″N, longitude 1°31′28″W) into 500 ml glass sample bottles containing sodium citrate, as an anticoagulant. Centrifugation separated platelet-rich plasma (PRP) from the citrated sheep whole blood (Heraeus Biofuge primo centrifuge, Germany) for 15 minutes at 3,000 rpm, four hours after obtaining the blood sample [16].

### 2.8. The Wound-Healing Effect of CFHE

We investigated the wound-healing effect of CFHE using the excision wound model [17] with slight modification. Briefly, Albino rats were anaesthetized using an injection of 40 mg/kg pentobarbitone intraperitoneally. The expected area of the wound to be created was outlined on the back of the rats on the dorsal thoracic region, 1 cm away from the vertebral column, and prepared by shaving the dorsal fur with Kleen Shave stainless steel razor blade while rubbing with 70% ethanol. Full-thickness circular excision wounds, sized about 30 mm, were created along the markings using sterilized toothed forceps, scalpel, and scissors. We achieved hemostasis by blotting the wounds with a cotton swab soaked in normal saline. The entire wound kept open. The rats were then grouped into A–E (*n* = 5) and measured the diameters of the excised wounds with a millimeter rule. Wounds of animals in Group A (Negative control) were treated with Simple Ointment BP, whereas those in Groups B, C, and D were treated 5, 10, and 15% w/w CFHE ointments, respectively. Group E (positive control) was treated with Silverzine (1% silver sulfadiazine cream; Ayrton Drug Manufacturing Ltd, Ghana), the reference drug. The ointment was topically administered for 14 days. The diameters of the excised wounds were measured on days 0 (the day we created the wounds), 3, 6, 9, and 12 postinjury, and the wound areas were calculated.

### 2.9. In Vitro Platelet Aggregation Test

Two-hundred-microliter (200 *µ*L) volumes of PRP each were mixed with 1 *µ*L of CFHE of concentrations of 0.8, 1.6, 3.2, 6.4, 12.5, 25, 50, and 100 mg/mL. Twenty microliters (20 *µ*L) of 0.1 m adenosine diphosphate (ADP) solution was added to each mixture as an aggregating agent and allowed 5 minutes for aggregation to occur. Absorbances of the resultant mixtures (*As*) were determined spectrophotometrically at a wavelength of 600 nm, and the results were presented as percentage inhibition of aggregation [18] using the following formula;

(1) $$\begin{array}{c} 1-\left(\frac{As}{A\;d}\right)\times 100. \end{array}$$

A similar test was done using 1% dimethyl sulfoxide (DMSO) solution (used as a negative control for this test), and the absorbance (*Ad*) was measured [19, 20]. The IC_50_ for CFHE was estimated. We carried the experiment out in triplicate.

### 2.10. Histological Studies

Wound tissues were aseptically removed on day 14 of the experiment on animals from each group and fixed in 10% formalin solution. The fixed tissue was prepared as described by [21]. We then observed the mounted glass slides under the light microscope (Leica Microsystems, Wetzlar, Germany) under × 40 magnification for changes and angiogenesis, cell repair, reepithelialization, collagen content, and granular tissue formation. Quantification of reepithelization, fibroblast proliferation, angiogenesis, and collagen deposits for assessing the wound healing in Albino rats was 1 = absence, 2 = slight, 3 = moderate, and 4 = extensive. Two researchers did the scoring in a blinded fashion, and the scores were collated and analyzed.

### 2.11. Anti-Inflammatory Activity of CFHE

The experiment was to study the anti-inflammatory activity of CFHE using the carrageenan-induced thigh inflammation previously described by Roach and Sufka with slight modification [22]. Inflammation was induced in 7-day-old cockerel by injecting carrageenan (10 *μ*L of a 2% solution in saline) into the subplantar tissue of the right foot after measuring their initial thigh thicknesses (*Ti*) using a digital caliper (Jiangsu BC Magnets Co. Ltd, China). We took again thigh thicknesses after 3-hour postcarrageenan injection (*T*0), and the inflammation was quantified. Chicks with thigh thickness >50% of the initial thickness were considered for the study. The chicks with inflamed thighs were randomly put into five groups A–E (*n* = 5). Group A, the negative control, was treated with Simple ointment BP. Groups B, C, and D were treated with 5, 10, and 15% *w*/*w* CFHE ointment. Group E, the positive control, was treated with Olfen Gel (1% diclofenac sodium ointment; Acino, Switzerland). All treatments were done topically with a mild massage. To prevent the drug from wiping off the inflamed area, the thigh was gauze-bandaged. We then measured thigh thicknesses at hourly intervals (*Tt*) over 6 hours, and then on the 24^th^ hour. The percentage decrease in thigh thickness was estimated using the following formula:

(2) $$\begin{array}{c} \frac{\left(Tt-\left[T0-Tt\right]\right)}{Ti}\times 100. \end{array}$$

We then plotted the percentage decrease in thigh thickness against time to establish the anti-inflammatory activity of CFHE.

### 2.12. Analgesic Activity of CFHE

Analgesic activity of CFHE was determined as described by Woode et al., in the Randell–Selitto pressure test [23] with slight modification. In this test, the initial pain threshold (*Pi*) in the 7-day-old cockerel was measured using the Randell–Selitto an algesimeter (Model: PPA01; Orchid Scientific and Innovative India private Ltd, India). Inflammation and, therefore, pain were induced in the right thigh of the cockerel as described under “anti-inflammatory activity of CFHE,” Cockerels with inflamed thighs were put into 3 groups (A, B, and C), and the pain threshold (*P*0) was again determined after 3 hours postinflammation induction. Chicks in Group A (the negative control) were treated with simple ointment BP. Group B was treated with 15% *w*/*w* CFHE ointment, and Group C (the positive control) was treated with Olfen Gel (1% diclofenac sodium ointment). Pain threshold (*Pt*) was measured at hourly intervals for 4 hours, and at daily intervals for 4 days. The percentage, increased in pain threshold (indicating analgesic effect) measured as the force applied by the algesimeter on the inflamed thigh to cause the withdrawal of the thigh from the algesimeter, was calculated by

(3) $$\begin{array}{c} \frac{\left(Pt-\left[P0-Pt\right]\right)}{Pi}\times 100. \end{array}$$

The percentage increased in pain threshold was then plotted against time to establish the analgesic activity of CFHE.

### 2.13. Antimicrobial Activity of CFHE

We investigated the antimicrobial activity of the CFHE using the Agar Diffusion Method. Nutrient agar (Oxoid Limited, United Kingdom) media were used for both determinations of the antibacterial activities. A 0.1 ml quantity of 18 hour culture of the test organisms (*Salmonella typhi*, *Escherichia coli*, *Pseudomonas aeruginosa*, *Staphylococcus. Aureus*, *and Streptococcus pyogenes*) was used to seed nutrient agar plates. In each of these plates, a well (10 mm) was cut out using sterile number 5 cork borer and was filled with 200 *µ*L each of 15% CFHE and allowed to diffuse at room temperature for 1 hour. We measured the zones of inhibition after 24 hours incubation at 37°C [24]. We carried the experiment out in triplicate.

### 2.14. Statistical Analysis

We analyzed data using Graph Pad Prism for Windows version 8 (Graph Pad Software, San Diego, CA, USA) and expressed values as Mean ± SEM. Significant differences between treatments and the control were determined using One-Way Analysis of Variance, followed by Dunnett's Multiple Comparisons Test. *p* ≤ 0.05 was considered to be statistically significant.

## 3. Results

### 3.1. The Wound-Healing Effect of CFHE

**Figure 1:**
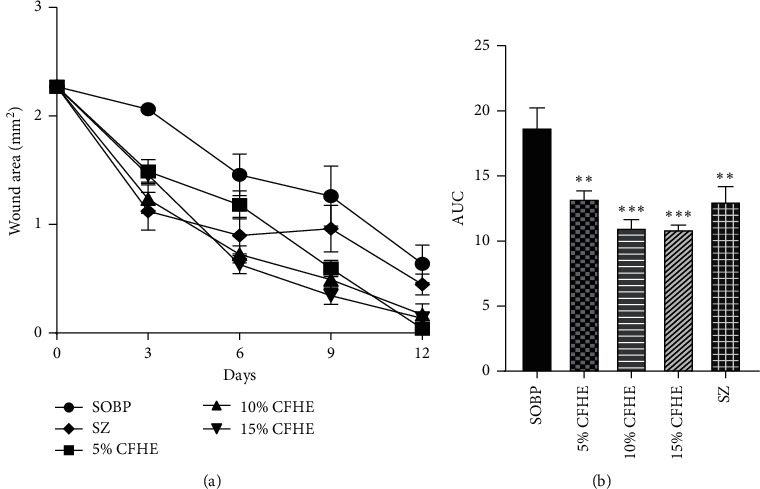
Effects of various concentrations of CFHE ointment, silverzine, and simple ointment BP on wound area in Albino rats. (a) Curves for time course of treatment, and (b) area under curve (AUC) of time-course curve. Values plotted are mean ± SEM, *n* = 5. ^*∗∗∗*^*p* ≤ 0.001; ^*∗∗*^*p* ≤ 0.001; treatment groups compared to control group (one-way ANOVA followed by Dunnett's multiple comparisons test).

All concentrations of CFHE ointment significantly (*p* ≤ 0.0001) reduced wound area from days 3 to 14 posttreatment compared to the simple ointment BP treated wounds. The effects observed were similar to those caused by Silverzine treatment (Figure 1).

### 3.2. Effects of CFHE on Platelet Aggregation

**Figure 2:**
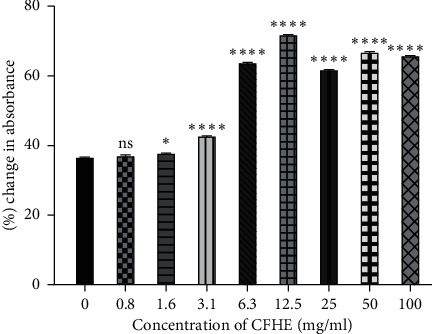
The percentage change in absorbances obtained for the different concentrations of the CFHE in an *in vitro* platelet aggregation test. Values plotted are Mean ± SEM (*n* = 3). Ns *p* > 0.05, ^*∗*^*p* ≤ 0.05, ^*∗∗∗∗*^*p* ≤ 0.0001, treatments compared to control group (one-way ANOVA followed by Dunnett's multiple comparisons test).

We observed that CFHE significantly (*p* ≤ 0.0001) promoted platelet aggregation at all concentrations used except at 0.8 mg/ml. We estimated the IC50 3.1–6.2 mg/ml (Figure 2).

### 3.3. Histological Evaluation of Treated Wounds

**Figure 3:**
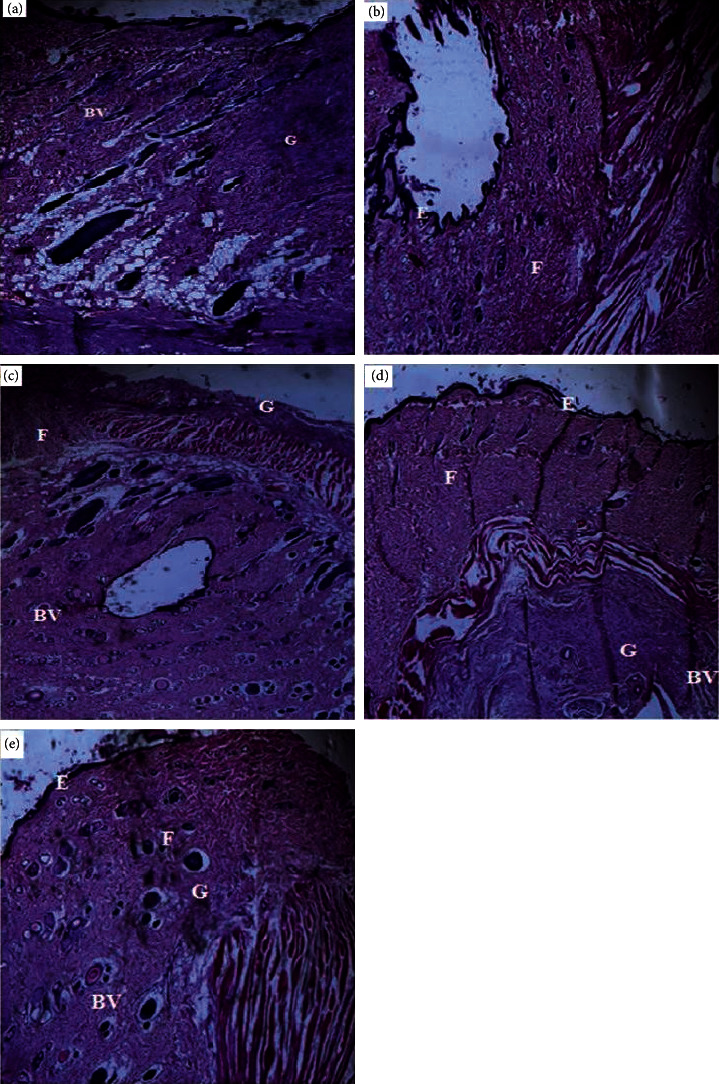
Sample photomicrographs showing effects of: (a) simple ointment BP, (b) 5% CFHE ointment, (c) 10% CFHE ointment, (d) 15% CFHE ointment, and (e) silverzine on excised scar tissues from Albino rat. BV = blood vessels, *E* = epidermal cells, *G* = granulation, *F* = fibroblast proliferation. Hematoxylin and eosin staining (× 400).

**Figure 4:**
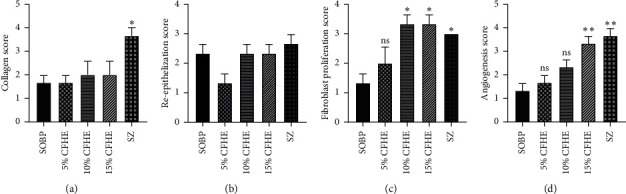
Effects CFHE ointment treatment on: (a) collagen deposits, (b) reepithelization, (c) fibroblast proliferation, and (d) angiogenesis in the wound-healing process in Albino rats. Values plotted are Mean ± SEM, (*n* = 5). ^*∗∗*^*p* ≤ 0.01; *p* ≤ 0.05 treatments compared to control group (one-way ANOVA followed by Dunnett's multiple comparisons test).

Histology of excised wound tissues showed improved wound-healing activity (i.e., fibroblasts proliferation, neovascularization, epithelial regeneration, and collagen deposition) with 15% CFHE and Silverzine-treated wounds compared to the control group (Figure 3). There were persistent inflammation, tissue necrosis, and less angiogenesis on treatment with simple ointment BP. However, treatment with 10 and 15% CFHE significantly (*p* ≤ 0.05) enhanced fibroblast proliferation and angiogenesis (Figure 4). Also, treatment with 1% sulfadiazine significantly (*p* ≤ 0.05) caused massive collagen deposition complemented with fibroblast proliferation and angiogenesis.

### 3.4. Anti-Inflammatory Effect of CFHE

**Figure 5:**
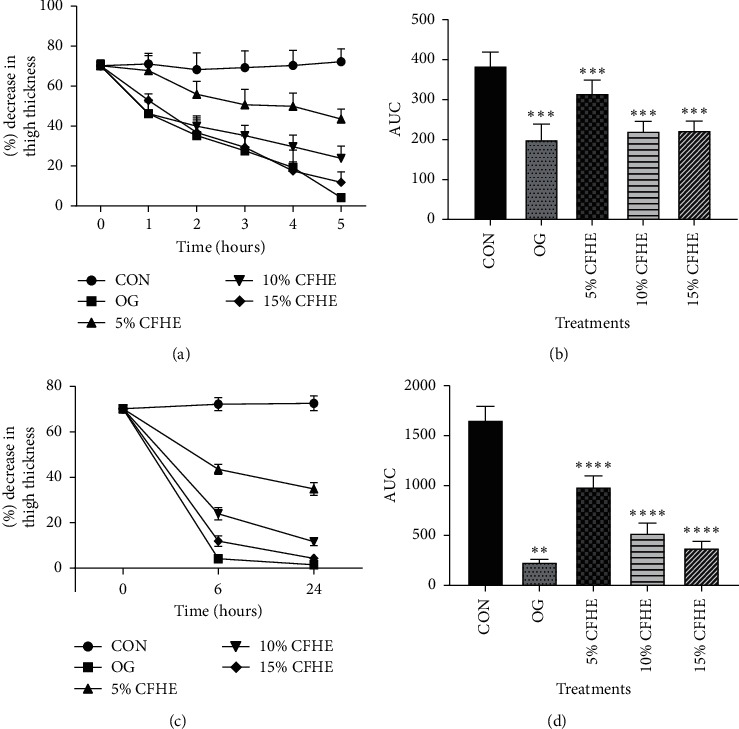
(a) The time-course curves for the effect of 5, 10, and 15% CFHE ointment and olfen gel (OG) on thigh edema in 7-day-old cockerels for 1–5 hours of treatment, (b) AUC for time-course curves in (a), (c) the time-course effects for effect of 5, 10 and 15% CFHE and 1% Diclofenac ointments on thigh edema in 7-day-old cockerels for 6 and 24 hours of treatment, and (d) AUC for time-course curves in (c); values are means ± SEM (*n* = 5). ^*∗∗*^*p* ≤ 0.01, ^*∗∗∗*^*p* ≤ 0.01 , comparing treatments versus control (CON); one-way ANOVA followed by Dunnett's multiple comparisons test.

Administration of carrageenan (10 *μ*L, 2% suspension) induced moderate inflammation resulting in thigh oedema in the 7-day-old chicks peaking at 2-3 hours. The CFHE ointment and Olfen gel significantly (*p* ≤ 0.0001) and dose-dependently inhibited carrageenan-induced oedema in chicks at all doses (Figure 5).

### 3.5. Analgesic Effect of CFHE

**Figure 6:**
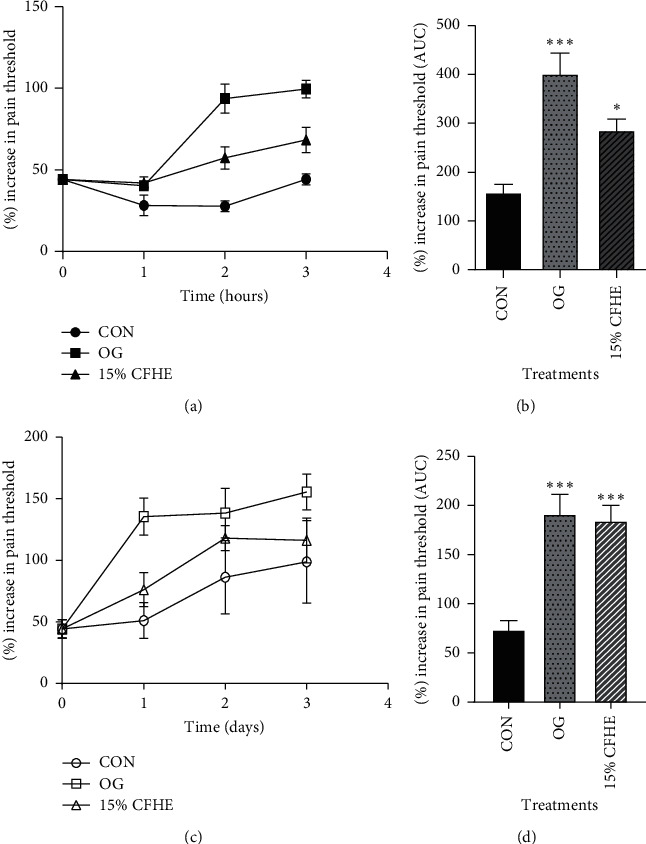
(a, c) The time-course effects of 15% CFHE ointment and olfen gel (OG) on pain management in hours, and in days, and (b, d) AUC for time-course curves for hourly and daily treatments, respectively. Values plotted are Means ± SEM. (*n* = 5). ^*∗*^*p* ≤ 0.05, ^*∗∗∗*^*p* ≤ 0.0001, comparing treatments versus control (one-way ANOVA followed by Dunnett's multiple comparisons test). CON = control.

A significant analgesic effect (*p* ≤ 0.05) was observed on the inflamed thigh of chicks treated with 15% CFHE and Olfen gel hourly for four hours, and daily for four days (Figure 6).

### 3.6. Antimicrobial Activity of CFHE

**Figure 7:**
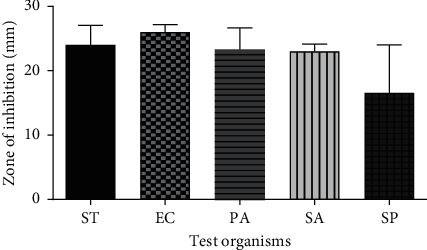
The inhibitory effect of 15% CFHE ointment on growth of *Salmonella typhi* (ST), *Escherichia coli* (EC), *Pseudomonas aeruginosa* (PA), *Staphylococcus aureus* (SA), *and Streptococcus pyogenes* (SP) in an agar diffusion test. Values are means ± SEM, (*n* = 3).

The 15% CFHE ointment was found to inhibit the growth of *Salmonella typhi*, *Escherichia coli*, *Pseudomonas aeruginosa*, *Staphylococcus. Aureus*, *and Streptococcus pyogenes* with the maximum zone of inhibition being 24 ± 5.3 and 26 ± 2.0 mm for *S. typhi* and *E. coli*, respectively. The minimum zone of inhibition was 16.57 ± 7.5 mm, which was for *S. Pyogenes*, while *P. aeruginosa*, and *S. aureus* were also inhibited with diameters of 23.3 ± 5.8 mm and 23 ± 2.0 mm, respectively (Figure 7).

## 4. Discussion

The aerial parts of *Cnestis ferruginea* are locally used in Ghana for treating various inflammatory conditions [25], so for wound management, a topical preparation (with varied concentrations) from the leaves of this plant was assessed for its wound-healing, platelet aggregation, and anti-inflammatory and analgesic, as well as antibacterial, effects.

The complexity of wound-healing processes involves the spatial and temporal synchronization of a variety of cellular substances with distinct roles in the phases of hemostasis, contraction, inflammation, granulation, collagenation, reepithelialization, and remodeling [1, 3, 26]. While hemostasis and inflammation characterized the inflammatory phase, the proliferative phase is complemented with epithelialization, angiogenesis, and deposition of collagen. A wound contract to reduce scar tissue, and this is seen with granulation [26]. Fibroblasts mostly appear in the wounds 72 hours after injury, and their accumulation requires phenotypic changes. The fibroblast in wounds proliferates profusely and produces matrix proteins such as hyaluronan, fibronectin, proteoglycans, and type 1 and type 3 procollagen [1]. Fibroblasts stimulate the production or synthesis of collagen, which is a major component of the extracellular matrix in organisms. Collagen production facilitates the expression of endothelial cells to promote angiogenesis, which enhances granulation of tissue formation and consequently argument wound healing, which is manifested as a decrease in the wound area. Wound closure of epithelial tissues occurs efficiently to restore rapidly the barrier function of the skin. The results from the excision model and the histopathological evaluation in Albino rats revealed that all concentrations of CFHE ointment exhibited significant wound healing promoting activity when applied topically. The effect was found to be concentration-dependent with 15% CFHE ointment, showing a more significant wound-healing activity by promoting reepithelization, fibroblast proliferation, formation of granulation tissue, deposition of collagen, and decrease wound area as compared to the simple ointment BP treated group. Hence, the significant reduction in wound area confirmed by histological studies showed that CFHE has an inherent wound-healing potential.

When blood vessels are damaged or injured, platelets rush and their binding facilitated form aggregates at the site of injury to the exposed activated thrombin receptor. Reepithelization of wounds is significantly accelerated in the presence of fibrin or platelet-rich plasma. Epithelization starts a few hours after injury and presents a single layer of cells accompanied by a marked increase in epithelial cell mitotic activity around the wound edges [1]. It involves blood platelets in normal homeostasis, which limits blood loss by regulating the interaction between components of the vessel wall, circulating blood platelets and plasma proteins. From the *in vitro* platelet aggregation test, CFHE showed an increment in platelet aggregation compared to the vehicle-treated samples. This may suggest that CFHE stimulates platelet aggregation and thus promotes epithelization. The ability of an agent to stimulate hasten wound-healing processes or exhibit antimicrobial, antioxidant, and anti-inflammatory activities is helpful [21] in the holistic approach to wound care management.

Carrageenan-induced oedema is commonly used as an experimental animal model for acute inflammation and is established to be biphasic [27]. Serotonin and histamine chiefly mediated the early phase (1 to 2 hours) of the carrageenan model and increased synthesis of prostaglandins in the damaged tissues. The late phase is sustained by prostaglandin release and mediated by bradykinin, leukotrienes, polymorphonuclear cells, and prostaglandins produced by tissue macrophages [27, 28]. CFHE significantly suppressed the inflammation induced by carrageenan in a curative protocol of the anti-inflammatory activity assessment. The various strengths of the CFHE ointment used showed significant activity on inflammation compared to the control. The study result for the time-course graph over 24 hours showed close to complete inhibition of inflammation. This suggests that the anti-inflammatory effect of the CFHE increases with time. The finding also justifies the use of the extract traditionally under the treatment of inflammatory conditions. The anti-inflammatory action of CFHE is consistent with a previous study by Singh et al. [29]. CFHE and Olfen gel exhibited significant analgesic activity in the carrageenan-induced pain model. Cutaneous inflammation releases endogenous proinflammatory mediators from damaged cells and aggregates them at the injured part of the body. This reduces the threshold of various mechanoreceptors and transduction mechanisms, thus producing hyperalgesia [30]. Therefore, it is possible that CFHE increases the threshold of cutaneous mechanoreceptors and/or decreases the production of pain endogenous mediators. But, in the analgesic test, a single dose of CFHE was employed. Therefore, the relative potency of the extract and diclofenac was not established.

CFHE from the study showed antimicrobial activity against *S. aureus*, S. pyogenes, *P. aeruginosa*, *E. coli*, and *S. typhi*, which are implicated in wound contamination and colonization [2, 31]. The presence of these microorganisms, inflammation, and oxidative stress retards wound-healing processes and consequently prolongs the early phase of wound healing [1, 32, 33]. Infected wounds become chronic and become difficult to treat. The antimicrobial activity of CFHE indicates its usefulness in the treatment and management of infected wounds.

The anti-inflammatory, antioxidant, analgesic, and antimicrobial activity exhibited by CFHE [29, 33, 34], contributing to its wound management effect in this study, could be attributed to phytochemical present in the plant. Secondary metabolites such as alkaloids, flavonoids, tannins, glycosides, terpenoids, and plant steroids found in various plant extracts handle their respective pharmacological properties [35, 36]. Tannins [37], flavonoids [38], saponins [39], and glycosides also enhance the wound-healing process based on their antimicrobial and antioxidant activities. Besides, we have identified glycosides to exhibit anti-inflammatory, as well as promote, wound healing. Parvez and Rahman [40] reported that an isoflavone glycoside afrormosia-7-O-beta-D-galactoside isolated from *C. ferruginea*, possesses broad antimicrobial activity. Thence, from preliminary phytochemical screening, the leaf extract of *C. ferruginea* contains secondary metabolites such as alkaloids, saponins, glycosides, tannins, and flavonoids [41] which may be responsible for its tradition in wound management.

## 5. Conclusion

Findings show that the hydroethanolic leaf extract of *Cnestis ferruginea* could be effective in the management of wounds, as it possesses wound-healing, platelet aggregation, anti-inflammatory, analgesic, and antimicrobial properties.

Further work could be done to assess some inflammatory mediators like serotonin, histamine, and prostaglandins in the damaged tissues in an early phase and bradykinin and leukotrienes in the late phase of inflammation; we further explain the mechanism of activity of the extract. Also, different doses of CHFE can be used in further analgesic studies to establish its relative potency.

## Data Availability

All data generated or analyzed during this study are included in this manuscript. However, the datasets used and/or analyzed during the current study are available from the corresponding author upon reasonable request.
